# Preparation of poly(*N*-vinylpyrrolidone)-stabilized ZnO colloid nanoparticles

**DOI:** 10.3762/bjnano.5.47

**Published:** 2014-04-03

**Authors:** Tatyana Gutul, Emil Rusu, Nadejda Condur, Veaceslav Ursaki, Evgenii Goncearenco, Paulina Vlazan

**Affiliations:** 1 Institute of Electronic Engineering and Nanotechnologies D. Ghitu, Academy of Sciences of Moldova, 3 Academiei str., Chisinau, MD-2028, Moldova; 2 State University of Republic of Moldova, 60 Alexe Mateevici str., Chisinau MD-2009, Moldova; 3 National Institute of Electrochemistry and Condensed Matter, 144 Dr. A. Paunescu Podeanu str., Timisoara 300569, Romania

**Keywords:** colloidal solutions, nanocomposite, poly(*N*-vinylpyrrolidone), sol–gel, zinc oxide

## Abstract

We propose a method for the synthesis of a colloidal ZnO solution with poly(*N*-vinylpyrrolidone) (PVP) as stabilizer. Stable colloidal solutions with good luminescence properties are obtained by using PVP as stabilizer in the synthesis of ZnO nanoparticles by a sol–gel method assisted by ultrasound. Nanoparticles with sizes of 30–40 nm in a PVP matrix are produced as a solid product. The colloidal ZnO/PVP/methanol solution, apart from the most intense PL band at 356 nm coming from the PVP, exhibits a strong PL band at 376 nm (3.30 eV) which corresponds to the emission of the free exciton recombination in ZnO nanoparticles.

## Introduction

Zinc oxide is widely used in various applications such as gas sensors, solar cells, antireflection coatings, varistors, surface acoustic wave devices, light emitting diodes and random lasers [1–4]. Among different processing methods, the sol–gel technique has various advantages such as cost-efficient processing, low-temperature sintering capability, the possibility of coating large and complex surfaces, and the capability to produce high quality coatings with a wide range of easily controlled thicknesses [5]. Preparation of ZnO nanoparticles by a colloidal method in the form of hydrosols was widely investigated in recent years in connection with a possible employment in biology [6]. ZnO nanoparticles have been synthesized in conjunction with different polymers such as polyethylene glycol (PEG) and poly(*N*-vinylpyrrolidone) (PVP). Nanoparticles of various morphologies were formed, and the photoluminescence intensity was increased because of the passivation of surface defects in the nanoparticles [7].

Nanohybrid films with resistivity of 10^8^ Ω·cm were obtained by using PVP with molar mass of 400,000 at various Zn^2+^/PVP ratios [8]. Colloidal solutions of ZnO are obtained by different methods. For instance, a nano-colloid has been synthesized using a top-down wet chemistry method with bulk ZnO powder with grain size of 1–2 mm as starting material [9]. Stearic or oleic acids have been used as capping agents in the stabilization technique to prevent agglomeration of the ZnO nanoparticles [9–11]. Colloidal ZnO solutions with nanoparticle sizes of 2–6 nm were also obtained with a method employing lasers [12]. The mechanism of nano-colloidal solution formation was previously investigated [13], while the production of colloidal solutions stabilized by polyvinylpyrrolidone was not previously described. In this context, the investigation of mechanisms for the formation of colloidal solutions and solid phases of ZnO nanoparticles by a colloidal method in the presence of poly(*N*-vinylpyrrolidone) (PVP) is of great interest. We propose in our work a method for the synthesis of colloidal ZnO solutions with PVP as stabilizer.

## Experimental

### Synthesis of colloidal ZnO solutions and nanocomposites

The following chemical were used in the synthesis processes: zinc acetate Zn(Ac)_2_·H_2_O (Aldrich, 99%); KOH (Aldrich, 99.0%); poly(*N*-vinylpyrrolidone) PVP10, *M*_S_ = 10,000 (Aldrich, 99%); methanol 99.9%; ethanol anhydrous (Sigma Aldrich); hexane (Aldrich, 99%); acetone (Sigma 99%). We have modified the typical synthesis of ZnO colloidal solutions [13]. Zinc acetate dihydrate (Zn(OAc)_2_·2H_2_O) powder (0.439 g) was added to a KOH solution (0.02–0.04 M). Poly(*N*-vinylpyrrolidone) PVP was dissolved in methanol under stirring at room temperature and the ZnO solution was added to set various ratios of Zn:PVP (from 1:0.1 to 1:0.5 wt %). The prepared mixture was put into an Erlenmeyer flask and heated to 60 °C for 4 h under continuous stirring in an ultrasonic bath. In order to remove impurities from the white powder, it was precipitated and washed with absolute ethanol several times after cooling to room temperature. The excess surfactant, unreacted precursor, and high-boiling point solvents were removed by means of a solvent containing hexane, anhydrous ethanol, and acetone in the proportion of 2:1:5 [14]. Centrifugation with a solvent containing acetone and hexane was carried out for site-selective precipitation. Finally, the powder was dried at 150 °C overnight and characterized with regards to its structural, morphological and optical properties (sample 1). An amount of 100 mg of the prepared powder in the form of ZnO/PVP nanoparticles was resuspended in 30 mL of methanol and put in an Erlenmeyer flask and held at room temperature for 2 h under continuous magnetic stirring. The produced colloidal ZnO/PVP solution (sample 2) is stable for 3 months.

### Characterization methods

Powder X-ray diffraction (XRD) and infrared absorption spectroscopy (FTIR) were used to characterize the size, shape, structure, and composition of the colloidal solution and nanocrystals. The XRD analysis of products was determined by powder X-ray diffraction (XRD) PW 3040/60 X’Pert PRO diffractometer system, using Cu Ka radiation with (λ = 1.5418 Å) in the range of 2θ = 10–80° at room temperature. FTIR absorption spectra were measured with a PerkinElmer Spectrum100 FTIR spectrometer in the spectral range of 650–4000 cm^−1^ with a resolution of 2 cm^−1^ at a close-to-normal light incidence on the substrate at room temperature. Photoluminescence (PL) spectra of the ZnO nanoparticles in methanol were measured at room temperature with an MDR-23 spectrometer. The samples were excited by a nitrogen laser with wavelength of 337.1 nm.

## Results and Discussion

**Figure 1:**
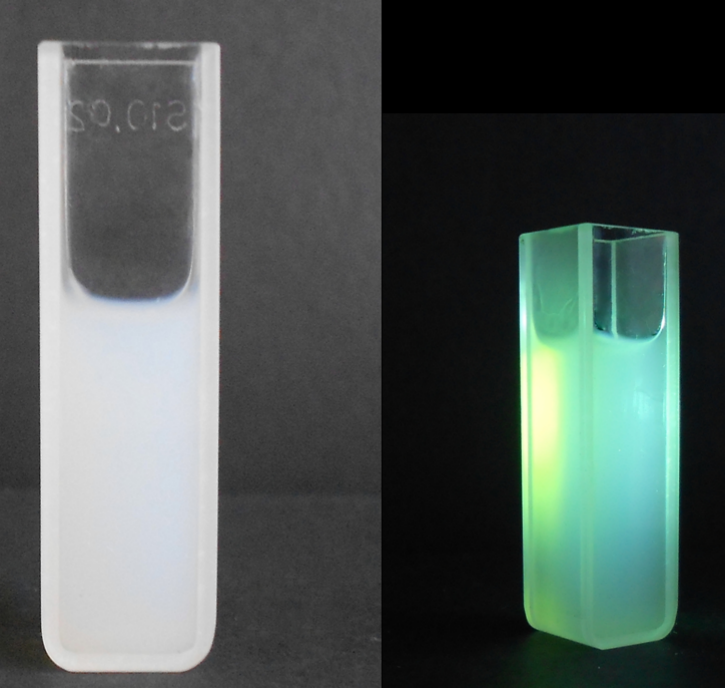
Photograph of colloidal ZnO /PVP nanoparticles (a), and luminescence under UV excitation with a HBO-103W/2 lamp (b).

The image of a cell with ZnO colloidal solution is shown in Figure 1. A bright luminescence is observed under the excitation of the solution with a HBO-103W/2 mercury lamp.

**Figure 2:**
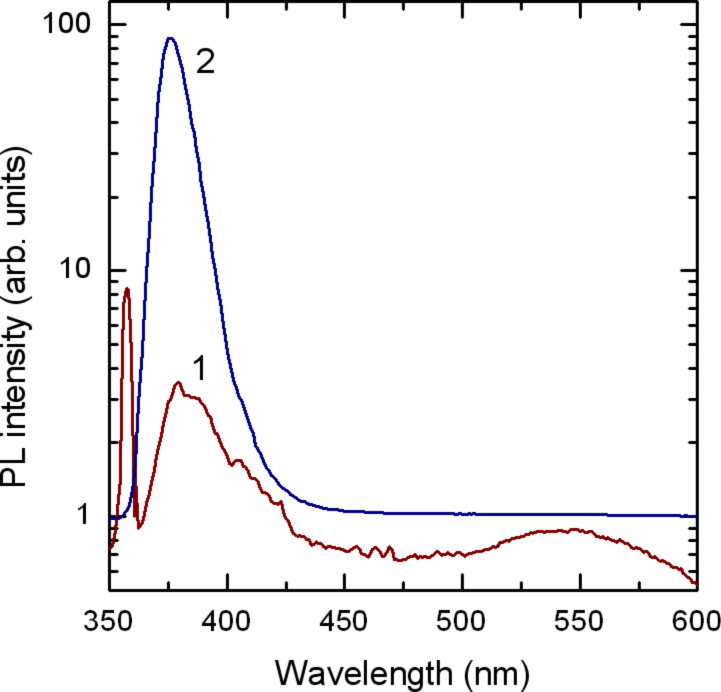
PL spectrum of PVP-capped ZnO nanoparticles (sample 1, curve 1) and PL spectra of ZnO bulk crystal (curve 2).

The PL spectrum of sample 1 compared to the spectrum of bulk ZnO crystals is presented in Figure 2. Apart from the emission band at 356 nm, which comes from the PPV shell, the spectrum of sample 1 exhibits ZnO near-bandgap luminescence with a maximum at 376 nm, which coincides with that observed in high-quality ZnO bulk crystals and is related to the recombination of free excitons. The observation of efficient free exciton emission at room temperature, as well as the weak visible emission observed at 550 nm is indicative of high-quality of ZnO nanoparticles in this sample, i.e., the PPV shell effectively passivates the surface defects of ZnO nanoparticles.

**Figure 3:**
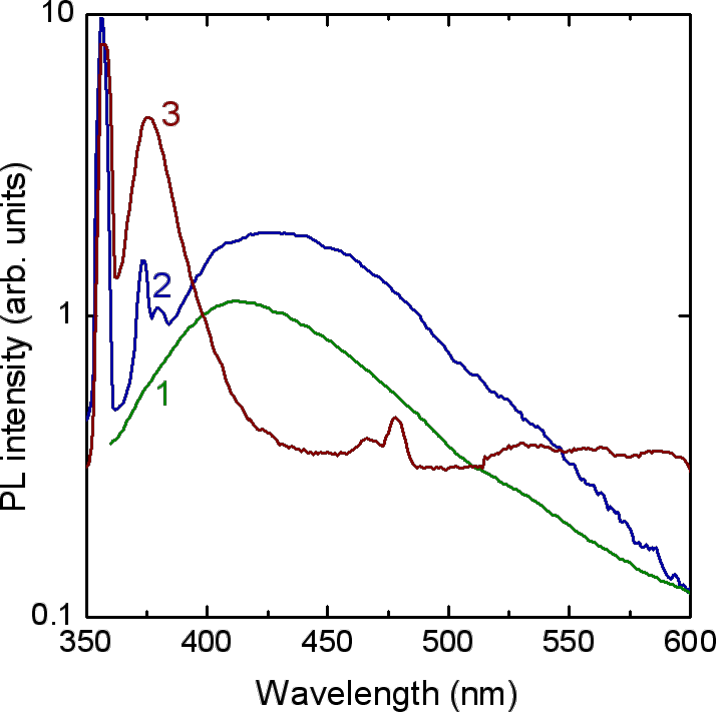
PL spectrum of methanol (1), PVP (2) and colloidal ZnO/PVP/methanol (3) at 300 K (Sample 1).

The photoluminescence spectra of the components of a colloidal ZnO/PVP/methanol solution are shown in Figure 3. The methanol exhibits a broad luminescence ranging from 350 to 600 nm. Two luminescence bands at 356 nm and 373 nm dominate the PL spectrum of PVP apart from a broad PL band with the maximum around 430 nm. A luminescence band around 370 nm has been previously found from PVP molecules [15], while a strong PL band was observed at around 350 nm in PVP-protected gold atomic clusters [16], and a broad luminescence band centered at 440 nm was found in PVP-capped ZnS nanoparticles [17]. The colloidal ZnO/PVP/methanol solution, apart from the most intense PL band at 356 nm coming from the PVP, exhibits a strong PL band at 376 nm (3.30 eV), which corresponds to the emission from the free exciton recombination in ZnO nanoparticles. The position of this emission band coincides with the one related to free exiton recombination in ZnO microwires [18]. Note, that if we will add the value of the free exciton binding energy of 60 meV to the position of this PL band (3.30 eV) we will obtain the value of the bulk ZnO bandgap at room temperature (3.36 eV). On the one hand, this is an indication for the high quality of the produced ZnO nanoparticles, since free exciton luminescence is observed only in highly pure material with a high structural quality. On the other hand, this hints at the absence of quantum confinement in the produced ZnO nanoparticles. The absence of quantum confinement effects is due to the relatively large size of the ZnO nanoparticles as compared to the exciton Bohr radius of around 2 nm in ZnO [19], as discussed below on the basis of XRD analysis. Note also that the emission in the visible spectral range, which is usually associated with structural defects, is practically absent from ZnO/PVP nanoparticles.

**Figure 4:**
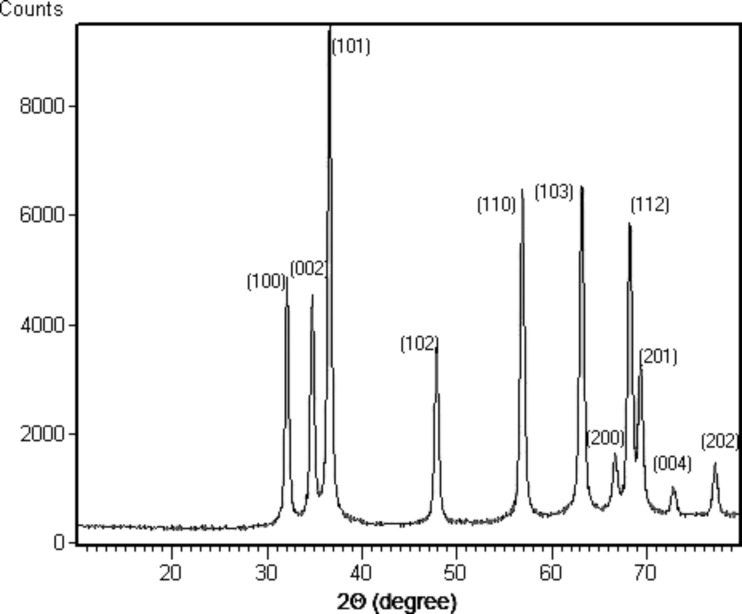
Typical powder diffraction pattern of ZnO/PVP NPs.

The XRD pattern of the powder material is shown in Figure 4. The diffraction peaks for 2θ = 31.71°, 34.40°, 36.42°, 47.58°, 56.84°, 63.12°, 66.52°, 68.12°, 69.18°, 72.58°, and 77.08° are observed, which correspond to the hexagonal phase of ZnO nanoparticles (space group *P*6_3_*mc*, *a* = 3.249 Å, *c* = 5.206 Å).

The size *d* of ZnO particles was estimated from the width of the most intense peak according to the Scherrer formula *d* = *k*·λ⁄(β·cos θ), where *k* is a constant, λ is the X-ray radiation wavelength (1.5418 Å), β is the full width at half maximum (FWHM), and θ is the diffraction angle. The particle size calculated from X-ray diffraction line width is around 30–40 nm.

**Figure 5:**
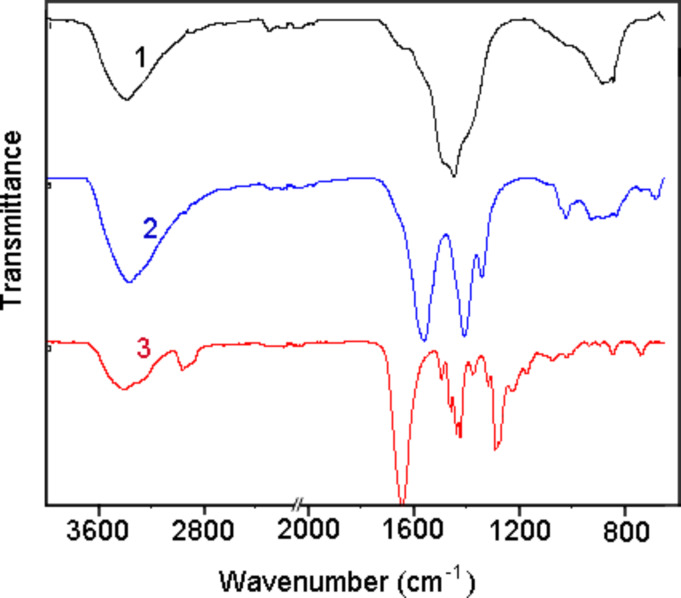
FTIR spectra of pure ZnO nanoparticles (1), ZnO/PVP composite (2), and pure PVP (3).

FTIR spectrum of ZnO nanoparticles (Figure 5) shows significant absorption peaks at 3377.6 cm^−1^, 1448.2 cm^−1^, and 882.2 cm^−1^. The band near 1448.2 cm^−1^ is assigned to H–O–H bending vibration mode due to the adsorption of moisture. The absorption band at 452 cm^−1^ assigned to Zn–O stretching vibrations, which was described in [20], was not observed in our experiments because of the limited capacities of the instrument.

The presence of various chemical functional groups in PVP (*M*_S_ = 10,000) is indicated by FTIR spectra as shown in Figure 5 (curve 3). The broad absorption band at 3399.6 cm^−1^ is due to O–H stretching vibrations of adsorbed water at the surface of particles. A peak at 1645 cm^−1^ is assigned to the stretching vibration of the C=O. The absorption peak at 1373.6 cm^−1^ is due to the С–Н bond in PVP [21]. Other important peaks at 1287.6 cm^−1^ and the doublet at 1437.8 cm^−1^, 1422.4 cm^−1^ are assigned to the C–N stretching vibrations and the attachment of CH_2_ groups in the pyrrole ring of PVP [22–23]. A comparison of the spectra of the prepared ZnO/PVP nanoparticles (Figure 5, curve 2) and pure PVP reveals similar absorption bands in regions of 3600–2400 cm^−1^ and 1650–650 cm^−1^. The peak observed in pure PVP at 1645 cm^−1^, which is due to С=О bonds, is red-shifted to 1559.6 cm^−1^ as a result of the interaction of the carbonyl oxygen with the zinc ion [23]. As concerns the absorption in the region of 1500–1200 cm^−1^, an overlap of absorption bands of PVP with bands of the hydrated zinc oxide, as well as interaction between them occurs. As a result, we observe a wide band with the maximum at 1406.5 cm^−1^ and a peak at 1341.3 cm^−1^. The first of these bands is related to the peak observed in ZnO nanoparticles at 1448.2 cm^−1^, which stems from the hydrated zinc oxide, but is shifted to 1406.5 cm^−1^, while the peak at 1341.3 cm^1^ is apparently attributed to the occurrence of covalent bonds of PVP with ZnO nanoparticles. Thus, our FTIR studies show that chemical reaction occur between the zinc oxide nanoparticles and polymeric stabilizer, by coordination of the zinc oxide particles with the nitrogen and oxygen atoms in PVP, as evidenced in [24].

## Conclusion

The usage of poly(*N*-vinylpyrrolidone) as a stabilizer in the synthesis of ZnO nanoparticles by an ultrasound-assisted sol–gel method allowed for the obtainment of stable colloidal solutions with good luminescence properties. The solid product consist of nanocomposite-encapsulated nanoparticles with sizes of 30–40 nm in a PVP matrix. The colloidal ZnO/PVP/methanol solution, apart from the most intense PL band at 356 nm coming from the PVP, exhibits a strong PL band at 376 nm (3.30 eV) which corresponds to the emission from the free exciton recombination in ZnO nanoparticles.
